# Ecological opportunity may facilitate diversification in Palearctic freshwater organisms: a case study on hydrobiid gastropods

**DOI:** 10.1186/s12862-018-1169-2

**Published:** 2018-04-19

**Authors:** Diana Delicado, Torsten Hauffe, Thomas Wilke

**Affiliations:** 1 Animal Ecology and Systematics, Justus Liebig University, Heinrich-Buff-Ring 26-32 (IFZ), D-35392 Giessen, Germany; 2 Department of Invertebrate Zoology, Smithsonian Institution, 10th and Constitution Ave, NW, Washington DC, 20013-7012 USA

**Keywords:** Speciation rate, Ecomorphological divergence, Disparity-through-time plots, Elevational gradients, Hydrobiidae, *Pseudamnicola*, *Corrosella*

## Abstract

**Background:**

Differences in species richness among phylogenetic clades are attributed to clade age and/or variation in diversification rates. Access to ecological opportunity may trigger a temporary increase in diversification rates and ecomorphological variation. In addition, lower body temperatures in poikilothermic animals may result in decreasing speciation rates as proposed by the metabolic theory of ecology. For strictly freshwater organisms, environmental gradients within a river continuum, linked to elevation and temperature, might promote access to ecological opportunity and alter metabolic rates, eventually influencing speciation and extinction processes. To test these hypotheses, we investigated the influence of environmental temperature and elevation, as proxies for body temperature and ecological opportunity, respectively, on speciation rates and ecomorphological divergence. As model systems served two closely related gastropod genera with unequal species richness and habitat preferences – *Pseudamnicola* and *Corrosella*.

**Results:**

Lineage-through-time plots and Bayesian macroevolutionary modeling evidenced that *Pseudamnicola* species, which typically live in lower reaches of rivers, displayed significantly elevated speciation rates in comparison to the ‘headwater genus’ *Corrosella*. Moreover, state-dependent speciation models suggested that the speciation rate increased with decreasing elevation, supporting the ecological opportunity hypothesis. In contrast, a significant effect of environmental temperature, as proposed by the metabolic theory of ecology, could not be observed. Disparity-through-time plots, models of ecomorphological evolution, and ancestral habitat estimation showed for *Pseudamnicola* species rapid morphological divergence shortly after periods of elevational and habitat divergence. In contrast, *Corrosella* species did not deviate from null models of drift-like evolution.

**Conclusion:**

Our finding that speciation rates are correlated with elevation and ecomorphological disparity but not with environmental temperatures suggests that differences in ecological opportunity may have played a key role in *Corrosella* and *Pseudamnicola* diversifications. We propose that *Pseudamnicola* lineages experienced higher ecological opportunity through dispersal to new locations or habitats in lowlands, which may explain the increase in speciation rates and morphological change. In contrast, the evolution of *Corrosella* in headwaters is likely less facilitated by the environment and more by non-ecological processes.

**Electronic supplementary material:**

The online version of this article (10.1186/s12862-018-1169-2) contains supplementary material, which is available to authorized users.

## Background

The search for the cause(s) of variation in species richness across biota has challenged biologists for centuries (e.g., [1–3]). Ultimately, the diversification rate (i.e., speciation minus extinction rate) and its change over time determine the richness of a group [4]. The simplest diversification model considers a constant rate of diversification over time and across lineages. Accordingly, species richness varies solely as an exponential function of taxon age, regardless of factors other than time (i.e., time-for-speciation hypothesis). Hence, old groups are expected to be more species-rich than younger groups, simply because they have had more time to diversify [5]. Additionally, differences in species richness across biota may be due to variation in diversification rates [3, 6]. Numerous studies have evidenced that a greater ecological opportunity can stimulate species diversification and ecomorphological divergence among taxa (e.g., in geckos [7] or rodents [8]). Ecological opportunity results from environmental change or colonization of novel habitats (favored by, for example, habitat connectivity) [9, 10]. Moreover, increased body temperatures and thus metabolic rates may cause an increase in diversification rates, as suggested by the metabolic theory of ecology [11].

Environmental gradients of habitat heterogeneity, −connectivity, or temperature, linked to factors such as elevation or latitude, for example, offer the opportunity to diversify. Although differences in species richness along elevational gradients have been documented in several taxa [12–14], little is known about the effects of elevation and co-varying body temperatures on diversification rates [15, 16]. For strictly freshwater organisms, elevational gradients are related to the river continuum, featuring higher habitat connectivity and higher environmental temperatures in lower reaches. Additionally, the more likely presence of dispersal vectors in lower reaches, such as birds and fish [17, 18], might facilitate the colonization of novel habitats and territories in organisms with low dispersal capacity. This may eventually influence speciation and extinction rates.

Although elevation was previously used as a proxy to study the effect of habitat connectivity and ecological opportunity on lineage diversification [19, 20], the interplay among elevation, habitat type, environmental temperature, and ecomorphological divergence in driving diversification in freshwater organisms has rarely been shown. Such relationships are best inferred in groups in which the factor time can be excluded [21]. The closely-related gastropod genera *Pseudamnicola* Paulucci, 1878 and *Corrosella* Boeters, 1970 (family Hydrobiidae Stimpson, 1865) provide an ideal system to study the ecological opportunity-diversification relationship. This is due to the fact that the genus *Pseudamnicola* has a lower clade age (7 ± 2 Ma) but comprises more species (ca. 45) than the genus *Corrosella*, which is older (12 ± 3 Ma) but less species rich (13 species) [22]. With a common ancestral area on the Iberian Peninsula [22], only the species-rich genus *Pseudamnicola* was able to disperse eastward to other Mediterranean islands and peninsulas (see Glöer et al. [23] for general information on the distribution) and to colonize a greater diversity of habitats, such as springs, coastal streams, and lakes. Overall, *Pseudamnicola* species inhabit middle and low elevation sites of the river continuum [24], whereas *Corrosella* species are mainly confined to upper mountain springs of the Iberian Peninsula and southern France [22, 25]. Moreover, morphological and anatomical differences among species are more remarkable in the genus *Pseudamnicola* than in *Corrosella* [24, 26].

Given that clade age does not explain differences in richness (i.e., the time-for-speciation hypothesis), we tested the predicted influence of the metabolic rate and ecological opportunity on freshwater-gastropod diversification. Using a hierarchical set of analyses, we first inferred a multi-locus phylogeny comprising 30 species of *Corrosella* and *Pseudamnicola*, and estimated speciation rates for the two genera. We then tested the metabolic theory of ecology and ecological opportunity hypotheses. For this, we assessed whether environmental temperature and elevation, as proxies for body temperature and ecological opportunity, respectively, influence lineage-specific speciation rates. Finally, we integrated the phylogeny with morphological, anatomical, and habitat-preference traits to assess the potential effects of these factors on ecomorphological divergence. Such a phylogenetic approach might help understand how ecological opportunity and metabolic rates shape speciation and ecomorphological diversification in Palearctic freshwater gastropods. It may also provide a baseline for revealing the drivers of diversification across freshwater organisms.

## Methods

### Materials studied

We compiled morphological, ecological, and DNA sequence data from a total of 202 representatives of 12 *Corrosella* and 18 *Pseudamnicola* taxa (Additional file 1: Table S1). Species delineation was based on Delicado et al. [22, 25] and on the genetic, morphological, and ecological data provided in the present paper (see Additional file 3: Table S2). Moreover, the inclusion of individuals from the type localities of *P. lucensis* and *P. calamensis* (Additional file 1: Table S1), helped clarify the identity of some *Pseudamnicola* species named as “sp. 1” and “sp. 3”, respectively, in the previous work of Delicado et al. [22]. In total, we included nearly 50% of the recognized species richness of the subfamily Pseudamnicolinae. However, our coverage is presumably higher since nearly half of the *Pseudamnicola* taxa available in the literature are described based on shell features only. For instance, due to shell convergence among several hydrobiid genera, some *Pseudamnicola* species might have been erroneously assigned to this genus [27].

### DNA sequencing

DNA sequences of two mitochondrial and one nuclear region from 194 specimens of *Pseudamnicola* and *Corrosella* were obtained from GenBank [22, 24, 26–28]. In addition, 8 specimens of *Pseudamnicola* were newly sequenced herein (for GenBank accession numbers see Additional file 1: Table S1). We amplified total DNA following the CTAB protocol of Wilke et al. [29]. Two mitochondrial fragments comprising 1168 base pairs (bp), including the cytochrome coxidase subunit I (COI) and the large subunit rDNA (16S), as well as 1043 bp of the nuclear large subunit rDNA (28S) were amplified using the following primers: LCO1490 and HCO2198 [30] for the COI fragment, 16Sar-L and 16Sbr-H [31] for 16S, and F63.2 and LSU3 for 28S [32, 33]. Previous studies on hydrobiid taxa showed that these fragments provide useful phylogenetic resolution at the species and/or genus levels [22, 34, 35]. All PCR amplifications were performed as in Delicado et al. [25] with annealing temperatures of 48 °C (COI), 50 °C (16S), and 51 °C (28S). Cycle sequencing products were analyzed on an ABI 3730 XL sequencer (Life Technologies, Carlsbad, CA, USA) using the Big Dye Terminator Kit (Life Technologies).

### Species tree inference

Bi-directional sequences were aligned in BIOEDIT v.7.5.3 [36] and compiled to gene-specific datasets. Alignments of the 16S and 28S fragments were conducted using the MAFFT multiple alignment program [37] with default settings for gap penalties (Gap opening penalty (GOP) = 1.53). Sequences of the protein-coding COI gene were unambiguously aligned in BIOEDIT.

The COI, 16S, and 28S data partitions were analyzed using Bayesian and coalescent methods, with and without outgroups, respectively (Additional file 1: Table S1). Prior to the phylogenetic analyses, we identified gene-specific nucleotide substitution models in JMODELTEST v.2.1.4 [38] under the corrected Akaike’s information criterion (AICc; [39–41]). The selected models were HKY [42] +I (invariable sites) +G (rate variation among sites) for the COI partition, K80 [43] +G for the 16S partition, and TIM3 [44] +I +G for the 28S partition in the presence of outgroups. Models TPM3uf [45] +I +G, K80 +G, and TrN [46] +I +G were selected for the COI, 16S, and 28S partitions, respectively, in the absence of outgroups.

To infer species relationships and assignments, we first conducted different Bayesian Inference (BI) analyses with the individual and concatenated datasets (including outgroups) in MRBAYES v.3.1.2 [47, 48] and the best-fit nucleotide substitution models through 2 independent runs of 4 Metropolis-coupled chains with 5 million generations each and a sampling frequency of 1000. After ensuring stationary of the chains (i.e., standard deviation of split frequencies below 0.01), we discarded the initial 10% of the trees as burn-in. The majority-rule consensus tree obtained in the combined analysis is depicted in Additional file 2.

We then generated an ultrametric species tree without outgroups in the program *BEAST [49] with the best-fit nucleotide substitution models inferred above. As these models are not available in the *BEAST interface BEAUti v.1.8.0 by default [50], they were specified manually. Because of the lack of a robust fossil record in the subfamily Pseudamnicolinae, the subsequent phylogenetic analysis was done using relative divergence time (i.e., all branches evolving with a rate of 1 substitution per site per unit of time). A birth-death model [51] was selected as prior topology, which is appropriate for species-level phylogenies. We ran 75 million generations, sampling every 2,000th tree. Resulting log files were checked in TRACER v.1.6 [52] in order to ensure that the posterior distribution of the parameters reached stationary (effective sample size, ESSs, above 200). The final species tree (i.e., the maximum clade-credibility tree, MCC tree) was identified in TreeAnnotator v.1.8.0, with the initial 10% of the topologies discarded as burn-in and displayed using FigTree v.1.3.1 [53].

### Dynamics in diversification rates

As explorative analysis, we first visualized the overall trend of lineage diversification in each genus by a lineage-through-time (LTT) plot of the MCC tree. We included the effect of phylogenetic uncertainty by calculating the 95% confidence interval of the LTT based on 1000 random post-burn-in trees of the *BEAST posterior distribution using the phytools 0.4-56 package [54] for the R 3.2 statistical environment [55]. Although these plots are frequently utilized to show general diversification trends, they do not inform about differences in evolutionary rates [4]. Therefore, a Bayesian analysis of Macroevolutionary Mixtures (BAMM; [56]) was performed utilizing the program BAMM v.2.5.0 [57], which models speciation and extinction trends over time. Because of the relatively low number of species and inherent limitations in inferring extinction rates from phylogenies [58], we here tested only for differences in speciation rates between clades. We accounted for an incomplete taxon sampling, which may bias rate inference [56], by including an analytical correction ratio of 0.4 for the genus *Pseudamnicola*. We sampled every 1000th out of 1 million BAMM generations, excluded the first 10% as burn-in, and inferred lineage specific speciation rates by averaging over the remaining posterior distribution of 900 generations. As suggested by Shi and Rabosky [59], the Bayes factor serves as measure of evidence for the number of rate shifts and integrates phylogenetic uncertainty. Accordingly, we subjected 1000 random post-burn-in trees of the *BEAST posterior to individual BAMM analysis, combined the BAMM posterior distributions into one pseudo-posterior, and calculated the Bayes factor to investigate whether there is statistical evidence for rate shifts among clades.

Recently, Moore et al. [60] indicated that the BAMM approach cannot detect rate shifts in completely extinct clades and is overly prior-sensitive (but see Rabosky et al. [57] for a reply). However, we did not infer extinction rates. Moreover, our analyses of the influence of elevation and environmental temperature on speciation rates confirmed the BAMM results (see below).

### Influence of environmental temperature and elevation on species diversification

We tested the influence of environmental temperature and elevation on speciation rates by Quantitative State Speciation and Extinction modeling (QuaSSE; [61]). QuaSSE models (1) the evolution of a trait (e.g., environmental temperature or elevation) by Brownian-motion (BM), and (2) the simultaneous influence of the trait-value on the probability of extinction or speciation through a trait-dependent birth-death process. Because hydrobiid snails are poikilotherm, water temperature may influence their occurrence and population growth [62, 63]. Water temperatures, in turn, have been shown to be correlated with annual air temperatures [64, 65]. Therefore, we extracted the mean annual temperature and the temperature of the warmest and coldest quarter for each sampled locality from the bioclim database [66] using the raster 2.6-7 package [67] for R. We calculated the occupied elevation of each species with elevation data provided by Delicado et al. [22, 25] and square-root transformed them to improve normal distribution. We used the average of the environmental temperature and elevation of all localities studied for each species (Additional file 3: Table S2) and a general standard deviation of 10% for the QuaSSE analyses. For the MCC tree and a random sample of 100 *BEAST post-burn-in trees, we used AIC-based model selection to identify the best fitting QuaSSE model out of 14 candidate models with increasing complexity. These models included separate sampling fractions for the two genera and for any or both genera constant or trait–dependent speciation rates. In addition, the QuaSSE analyses included environmental temperature or elevational evolution along the phylogenetic history, which was parameterized by equal or genus-specific rate of change (σ^2^) (for the different combinations see Additional file 1: Tables S4, S5). Since extinction rates are notoriously difficult to estimate based on molecular phylogenies [58], we did not test the influence of elevation on extinction.

### Morphological disparity and habitat evolution

To estimate morphological disparity over time, we measured 26 traits (20 continuous morphometric and 6 discrete morphological traits) from shell and radula as well as from the reproductive, respiratory, and nervous systems (see Additional file 1: Table S3 and Additional file 3). For the genera *Corrosella* and *Pseudamnicola*, we analyzed data from 233 and 172 shells, as well as 103 and 82 soft bodies, respectively (including data from Delicado et al. [24, 26, 28]). Sample sizes for each species ranged from 1 to 31 individuals (mean 10.7) for shell dimensions and 1–14 (mean 5.5) for anatomical structures. New dissections and measurements were made with a Keyence VHX-2000E digital microscope (Keyence Corporation, 2009-2012). We coded all discrete variables according to the standard nomenclature of morphological characters for hydrobiid snails [68] and made continuous variables size-independent by dividing them by shell length. Two variables, the average and standard deviation of elevational values within species (Additional file 3: Table S2), represented the occupied elevation of each species. No overlap in elevational ranges was observed between the genera, except for *P. subproducta*, whose upper boundary falls into the *Corrosella* range.

We estimated rates of morphological and elevational divergence as ratios of trait variation among subclades and the entire phylogeny through time (‘disparity’). The obtained disparity-through-time (DTT) [69] was compared with the disparity expected under the null model of trait evolution, the BM model. Disparity exceeding this expectation indicates a time period of rapid trait divergence. Because of the combination of continuous and discrete variables, we could not use the standard DTT approach of morphological data reduction via principle component analysis (e.g., [70]). Instead, we used the first two axes of a Gower’s distance [71] based on metric multidimensional scaling (MDS). With 30.5 and 15.1%, respectively, the first two axes explained more variance than expected by the broken-stick criterion and were retained for subsequent analyses. We performed separate DTT analyses for the two genera using the geiger 2.0.3 package [72] for R and then compared the morphological and elevational DTT plots for each genus.

After this explorative analysis, we tested whether rates of morphological divergence differed significantly between the two genera. Most phylogenetic comparative methods can handle only one continuous trait and have less power in comparison with approaches that include several morphological traits (e.g., [73–75]). We subjected the first two axes of the MDS jointly to competing multivariate phylogenetic comparative analyses and used AIC-based model selection to test for heterogeneity in rate of morphological evolution (σ^2^) between the two genera and the two MDS axes. Given the small size of our phylogeny, more complex evolutionary models, such as the Ornstein-Uhlenbeck model of morphological adaptation, could not be tested here. All four BM models (see Additional file 1: Table S6) were fitted for the MCC tree and a random sample of 100 *BEAST post-burn-in trees utilizing the OUwie package 1.5 [76] for R.

Finally, we investigated whether the species occurring at lower elevations, i.e., *Pseudamnicola* spp., were better able to colonize new habitats than those living at higher elevations. We computed ancestral habitat preferences assuming the same processes as in Matzke [77], i.e., habitat expansion (by adding a new habitat type to the descent, parameter *D*), habitat extirpation (descents with less habitat types than ancestors, parameter *E*), and founder effect (habitat-switching process during a cladogenetic event, parameter *j*). Under these assumptions, the estimation of habitat type was conducted along the MCC tree with the R package BioGeoBEARS 0.2.1 [78]. Using sample-size corrected AICc, we statistically compared the goodness-of-fit of the following models of habitat evolution: dispersal–extinction–cladogenesis (DEC; [79]), dispersal–vicariance (DIVA; [80]), and BayArea [81]. In order to calculate an AICc, we used the likelihood version (referred as DIVALIKE and BayAreaLIKE; [78]) of the latter two models. In addition, we tested for model-improvement by including the founder effect parameter *j* to each of these models. In order to avoid a large range of habitat states, we grouped the data in six general habitat categories: rheocrene spring, helocrene spring, mountain stream, coastal stream, lake or pond, and river. We set the maximum of ancestral habitat types to three, which is the maximum number of occupied habitats found in Pseudamnicolinae species. Moreover, we disallowed ecological unlikely combinations, such as mountain streams and coastal streams, which minimizes the total number of habitat states and uncertainty in the estimation of ancestral habitat type.

## Results

### Phylogeny and dynamics in speciation rates

**Fig. 1:**
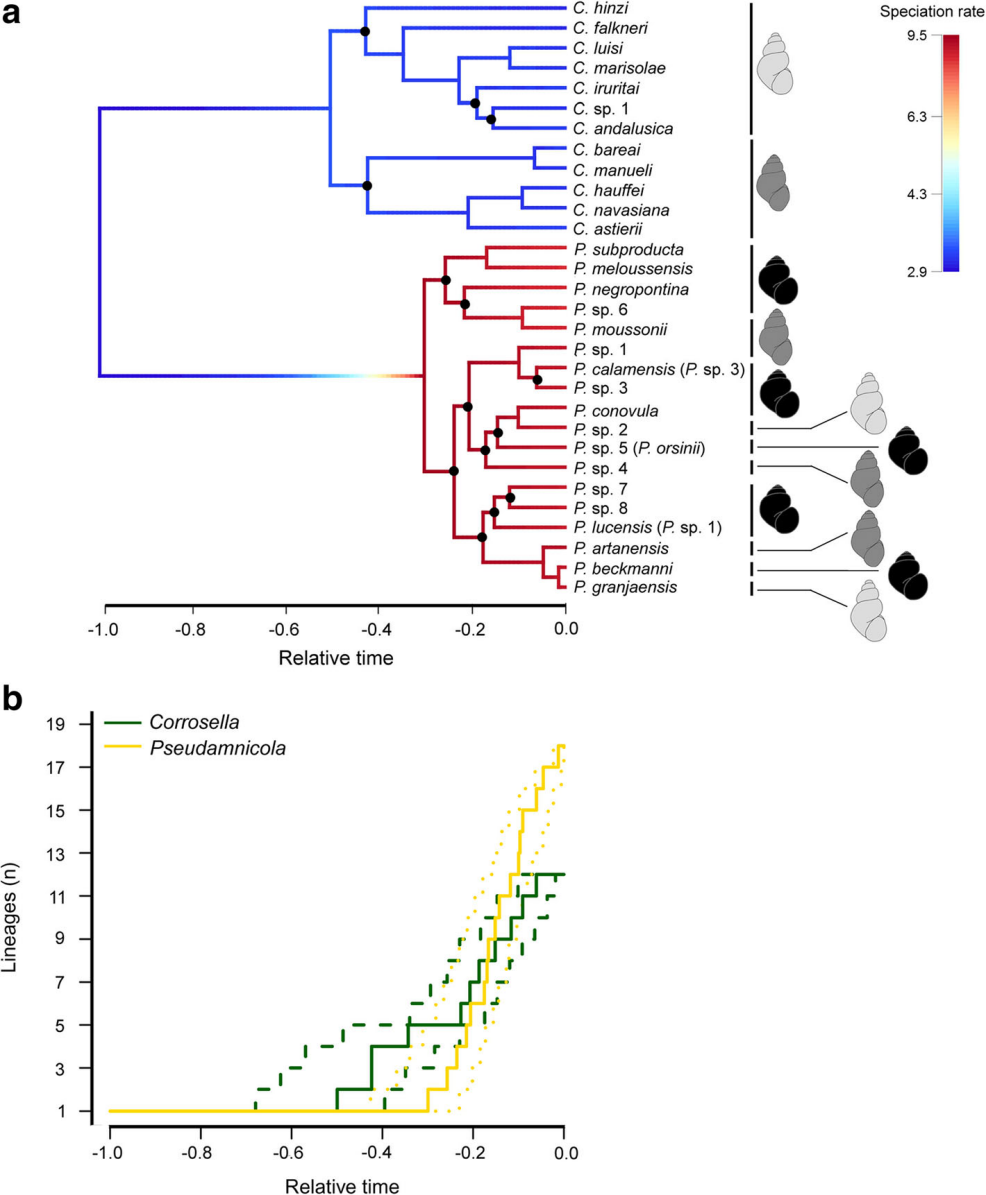
Speciation dynamics in the genera *Corrosella* and *Pseudamnicola*. (**a**) Maximum clade credibility tree computed in *BEAST with branch lengths proportional to relative time. Tips represent the species to which individuals were assigned. Species names in parentheses refer to the previous species assignments made in Delicado et al. [22]. Black dots on nodes indicate branches supported by BPP < 0.9. For the phylorate plot, branches were color-coded according to modeled speciation rates. Three categories of shell shapes were identified (shell figures), according to shell length / shell width ratios of 0.4–0.8, 0.9–1.3, and 1.4–1.9. (**b**) Linage-through-time plots of the MCC tree (solid lines) and the 95% confidence interval based on 1000 post-burn-in trees (dashed lines) are shown for each genus separately

The MRBAYES analysis of the concatenated dataset revealed 30 clades of reciprocally monophyletic *Pseudamnicola* and *Corrosella* populations (Additional file 2), which can also be distinguished morphologically (see morphological traits of Additional file 3). Ten *Pseudamnicola* and 11 *Corrosella* clades were assigned to known species and 9 to potentially new species (*Pseudamnicola* sp. 1–8 and *Corrosella* sp. 1) according to the phylogenetic species concept [82]. Though the BI analyses supported the monophyly of both *Corrosella* and *Pseudamnicola*, some species-level relationships, especially within the genus *Pseudamnicola*, remained poorly supported (Additional file 2). These weakly supported clades concur with a notable increase in the speciation rate at the root of *Pseudamnicola* (Fig. 1a). However, all new *Pseudamnicola* taxa were relatively well resolved in the trees based on the mitochondrial and concatenated datasets. Only in the tree based on the conservative 28S dataset, two species, *Pseudamnicola* sp. 3 and *Pseudamnicola* sp. 5, were weakly supported.

Our BAMM analysis, based on the MCC tree and 1000 post-burn-in posterior trees, revealed a Bayes factor of 8.5 upon comparing a speciation model including one rate shift with a model without shifts. According to Jeffreys [83], this indicates substantial support for one hypothesis over the other. With a Bayes factor of 1.1, a speciation model with two rate shifts is not better supported than the one with a single shift. The genus-specific macroevolutionary regimes are also reflected in the different branching patterns and in the steeper slope of the LTT plot for the *Pseudamnicola* clade (Fig. 1b).

### Influence of environmental temperature and elevation on diversification

The QuaSSE analysis did not reveal a significant relationship between speciation rate and mean annual temperature (Additional file 1: Table S4, Fig. S2). With a ΔAIC = 1.37 for the MCC tree and a mean rank = 3 for 100 random post-burn-in trees, the best temperature-dependent speciation model is outperformed by temperature-independent models. The best-fit model (ΔAIC = 0.00; mean rank = 1) includes genus-specific rates of speciation and temperature divergence (σ^2^).

**Fig. 2:**
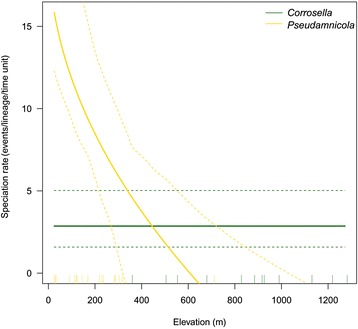
Relationship between elevation and speciation rates in the genera *Corrosella* and *Pseudamnicola*. A joint QuaSSE modeling of elevational evolution along the phylogeny and its influence on speciation revealed a decreasing speciation rate in *Pseudamnicola* with increasing elevation. We calculated the 95% highest posterior density (dashed lines) of speciation rates by Bayesian inference. Vertical ticks on the x-axis indicate elevation of extant species. Note that for model assumptions of normality, we took the square root of elevation. The back-transformation caused the non-linear impression of the plot

For the relationship between speciation rate and elevation, model selection favored a QuaSSE model (Additional file 1: Table S5) of genus-independent rates of elevational divergence (σ^2^). It also revealed a linear relationship between speciation rate and elevation in *Pseudamnicola* but not in *Corrosella*. For the former genus, lineages occupying lower elevation speciated at a higher rate than high-elevation lineages (Fig. 2). Taking into account the phylogenetic uncertainty based on 100 random post-burn-in trees, this scenario obtained the highest mean rank of 1.30 among all 14 QuaSSE models. The second best QuaSSE model received insignificantly lower support (ΔAIC = 0.93; mean rank = 2.66) but differed from the best model only by including additionally genus-specific rates of elevational evolution. In general, a model of genus-specific speciation rates outperformed the scenario of an equal rate (see Model 5 versus 1 in Additional file 1: Table S5), thus confirming the BAMM result.

### Ecomorphological evolution

**Fig. 3:**
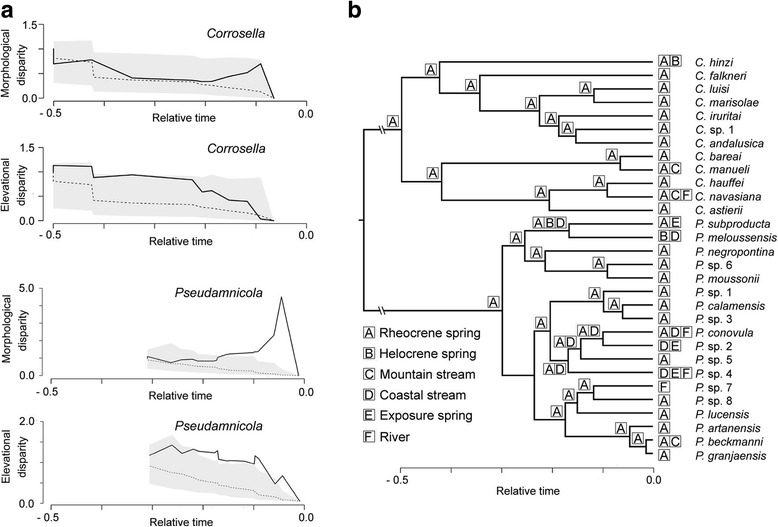
Ecomorphological disparity in the genera *Corrosella* and *Pseudamnicola*. (**a**) Morphological and elevational disparity-through-time plots displayed for *Corrosella* and *Pseudamnicola*. The black solid line indicates the observed disparity. The dashed line and gray area represent the mean and 95% confidence interval, respectively, of the expected disparity under a null model of morphological or elevational evolution along the phylogeny. (**b**) Estimation of ancestral habitat type through relative time performed in BioGeoBEARS for *Pseudamnicola* and *Corrosella*

The disparity-through-time plots showed different trends in the ecomorphological evolution of both groups. *Pseudamnicola* species experienced an abrupt increase in morphological disparity around 0.1 units of time ago (Fig. 3a), deviating significantly from the BM expectation. This disparity was already reflected in the variety of shell morphotypes found within some clades of *Pseudamnicola* (Fig. 1a). The elevational disparity of *Pseudamnicola* also deviated significantly from BM, predating the extraordinary morphological disparity by 0.15 time units (Fig. 3a). Morphological and elevation curves in *Corrosella*, on the contrary, showed similar trends through time with a gradual decline towards the present and no deviation to the expectation of the BM model of trait evolution (Fig. 3a).

The AIC-based comparison of competing models of morphological evolution (Additional file 1: Table S6) confirmed genus specific rates of morphological divergence. The best performing candidate for the MCC tree and 99% of all random post-burn-in trees included genus and trait specific rates of morphological divergence. For both trait axes, *Pseudamnicola* displayed significantly higher rates of morphological divergence than *Corrosella* (Additional file 1: Table S6).

In the BioGeoBEARS analyses, the dispersal-extinction-cladogenesis model with founder-event speciation (DEC + J) was favored (Table 1). Accordingly, the most recent common ancestor (MRCA) of both genera likely inhabited rehocrene springs (see Fig. 3b). The genus *Corrosella* expanded its habitat range only after the most recent speciation events. In contrast, the genus *Pseudamnicola* started to increase its habitat range during the second half of its phylogenetic history (i.e., relative time unit 0.15), which corresponds to the time of increased elevational disparity.

**Table 1:** Models of ancestral habitat evolution for *Corrosella* and *Pseudamnicola* species fitted by maximum likelihood in BioGeoBEARS

Model	P	D	E	J	ΔAICc
DEC + J	3	0.390	~ 0	0.006	0
DEC	2	0.428	0.272	–	1.01
BayArealike	2	0.622	0.750	–	5.68
BayArealike+J	3	0.392	~ 0	0.009	6.98
DIVAlike+J	3	0.415	~ 0	0.006	8.67
DIVAlike	2	0.467	0.047	–	13.54

Shown are the number of model parameters (P) and coefficients for range expansion (dispersal, D), contraction (extinction, E), and founder events (jump, j). Coefficients based on our phylogeny should not be compared with other studies because of our relative time calibration. Extinction coefficients of approximately zero indicate estimates at parameter bound of the model. We ranked the models according to the difference of model-fit in comparison to the best model (ΔAICc). The two best models do not differ substantially in their support (ΔAICc < 2) and showed similar ancestral habitat estimates

## Discussion

In this study, we tested the role of metabolic rate and ecological opportunity in freshwater-gastropod diversification, using two closely related gastropod groups that differ in species richness and environmental preferences. For doing so, we assessed the correlation of speciation rates with both environmental temperature (i.e., a proxy for body temperature linked to the metabolic rate) and elevation (i.e., a proxy for ecological opportunity). Moreover, we inferred the evolution of habitat types and morphological disparity along the phylogenetic history. We found fundamental differences in tempo and drivers of diversification between high- and low-elevation species. The increase in speciation rates towards lower elevations and the observed strong and rapid morphological divergence in *Pseudamnicola* species shortly after periods of elevational and/or habitat change suggest an important role of ecological opportunity in their diversification. In contrast, *Corrosella*, which is strictly confounded to high mountain springs, exhibited a lower rate of ecomorphological divergence probably driven by non-ecological processes, such as population fragmentation and allopatry.

By choosing an old but species-poor group and a young but species-rich group as model taxa, we were able to exclude a priori clade age as parameter affecting richness differences (i.e., the time-for-speciation hypothesis). Moreover, our QuaSSE analysis of linking environmental temperature and speciation rate did not support the metabolic theory. Thus, the variation in diversification rates observed (Fig. 1a) might be caused by phenotypic and/or ecological differences between these two groups. However, past ecological opportunity is notoriously difficult to evaluate [84], especially in groups lacking a robust fossil record. Nonetheless, phylogenetic comparative methods can be used to test for the signature of ecological opportunity, such as high speciation rates and morphological diversification upon colonizing new environments free of competitors [69]. Accordingly, the two following lines of evidence suggest that differences in ecological opportunity along the river continuum may have played a key role in *Corrosella* and *Pseudamnicola* diversifications.

First, the QuaSSE analysis (Fig. 2) showed that species occurring at mid and higher elevations (i.e., *Corrosella* species) are characterized by a constant diversification rate. Further, we found that diversification rates in *Pseudamnicola* species increased from mid to lower elevations, thus implying the transition to lower elevations as a potential cause for their extraordinary diversification. One possible explanation is that the higher connectivity among lower-reach localities (see Hughes et al. [85] for movement of freshwater faunas within a dendritic stream network), as well as the generally increased presence of dispersal vectors at low elevations, such as birds and fish [17], might have facilitated dispersal and colonization of novel habitats (also see [24, 35]). In fact, the estimation of ancestral habitat types indicated more habitat transitions during the evolutionary history of lowland species (i.e., *Pseudamnicola* species) than in headwater species (Fig. 3b).

Second, we observed that the origin of the strong morphological divergence among closely related species (i.e., positive deviation from the BM model of trait evolution) corresponded to the time of increase in their habitat diversity (Fig. 3a, b). Our ancestral habitat-use estimation showed a common habitat type (i.e., rheocrene spring) for the MRCAs of both groups and subsequent habitat switches and -extensions (e.g., from rheocrene springs to coastal streams) for some *Pseudamnicola* clades. Strikingly, the increase in morphological disparity occurred mainly upon divergence of those *Pseudamnicola* clades that showed greater habitat diversity, such as the clade formed by the species *P. conovula*, *Pseudamnicola* sp. 2, *Pseudamnicola* sp. 4, and *Pseudamnicola* sp. 5 (see Fig. 1a for shell morphology variation). Such a pattern of rapid morphological divergence as a response to ecological opportunity has been shown to be more common in young clades than during early phylogenesis [86]. In contrast, clades that tend to retain their ancestral habitat type, as for most *Corrosella* species, show phenotypic similarity among closely related taxa (Fig. 3a), as predicted by a BM model. Cases of low morphological variability among closely related species that share habitat preferences have been frequently reported in freshwater gastropods [87–89]. This is also true for patterns of high ecomorphological divergence associated with colonization of diverse habitats [90–92].

Morphological disparity and habitat divergence among closely related species are important operational criteria for evaluating the impact of ecological and non-ecological processes on speciation [93, 94]. The underlying assumption is that rapid habitat divergence, as a result of the colonization of novel environments free of competition and predation [95, 96], promotes also rapid ecological speciation and morphological disparity through natural selection [97, 98]. Well-known examples of ecological speciation are related to the colonization of emerging islands or lakes [98–100]. In our study, strong evidences for ecological speciation can be found in those *Pseudamnicola* clades, whose species inhabit distinct freshwater habitat types at low elevations within the river continuum. Alternatively, similar ecological preferences among closely related species (e.g., phylogenetic niche conservatism; [101]) together with geographical isolation may play a key role in non-ecological speciation [101–103]. *Corrosella* species are strictly confined to springs, which may reflect niche conservatism. In addition, these species present an allopatric distribution with small range sizes [25] and low morphological diversity (Fig. 3a). Both aspects have been often linked to non-ecological speciation [103–105].

Whereas in land snails [106, 107], and also freshwater snails [87], non-ecological speciation often seems to lead to a rapid lineage accumulation (defined as non-adaptive radiation; [105]), we found no evidence of faster evolution within *Corrosella* in comparison to *Pseudamnicola*. This leads us to hypothesize that: i) as ecological speciation is typically faster than non-ecological speciation [9], a potential increase of speciation rate in *Corrosella* could be concealed by ecological processes driving even faster divergence within the genus *Pseudamnicola*; ii) the higher degree of habitat specialization estimated in *Corrosella* species may have also influenced their extinction rates since habitat specialists are more vulnerable to habitat decline than generalist [108, 109], such as *Pseudamnicola* species. However, this second hypothesis could not be directly addressed because the small number of taxa in our phylogeny did not allow for the estimation of extinction rates. More comprehensive phylogenies linked to ecomorphological species traits are therefore needed for a better understanding of the role of ecological opportunity in driving tempo and mode of diversification (and ultimately in species richness) of freshwater biota.

### Limitations of this study

The integration of molecular, morphological, and ecological information from > 200 individuals of 30 freshwater gastropod species provided insight into the evolutionary history of species diversification in the subfamily Pseudamnicolinae. However, we acknowledge several limitations of our study.

First, not all known *Pseudamnicola* species were included, which could have biased the results of the morphological disparity analysis. As maximum disparity was found in young clades of closely-related species that occurred in areas intensely sampled, such as those from Mallorca Island (*P. beckmanni*, *P. granjaensis*, and *P. artanensis*) and Tunisia (*Pseudamnicola* sp. 2 and *Pseudamnicola* sp. 4), we believe that the inclusion of more species from unsampled areas is unlikely to change this result.

Second, phylogenetic comparative methods typically assume that phylogenetic relationships among species are correct, whereas our MCC tree (Fig. 1a) displayed a number of poorly supported nodes. However, macroevolutionary studies are usually robust against phylogenetic uncertainties [110]. Even though multiple branching events within a short period of time may not be resolved unambiguously, resulting in a low clade support, the speciation rate for this period is not affected per se. Moreover, our use of random samples of post-burn-in trees for phylogenetic comparative methods integrates this uncertainty [111].

Third, the diversification events inferred in the current paper are not time-calibrated due to the lack of respective calibration points derived from the fossil record and/or geological events. Thus, specific climatic or biogeographic events could not be directly correlated with the burst in speciation rate and habitat expansion seen in *Pseudamnicola*. However, this does not affect the inference of differences in tempo of species diversification between *Pseudamnicola* and *Corrosella,* as such differences can be well inferred with a relative-time approach [112].

Fourth, temperatures derived from global climatic databases are only a rough proxy for body temperature of poikilothermic animals and its influence on metabolic- and therefore speciation rates. Moreover, our sampling design was optimized for species coverage and not for spanning the full range of annual and intraspecific variations in in-situ water temperature but detecting an effect is unlikely with increasing variation (Additional file 1: Fig. S2). Thus, our inability of finding a significant relationship between environmental temperatures and speciation rates does not necessarily exclude a metabolic effect.

## Conclusions

By using two closely related groups of freshwater gastropods, we presented evidence indicating that speciation rates and ecomorphological divergence were higher in lowland than in highland species. In contrast, an influence of environmental temperature on the tempo of diversification processes could not be inferred. We therefore postulate that the higher connectivity among the lower reach localities as well as the more likely presence of birds and fish at low elevations may have facilitated access to ecological opportunity for *Pseudamnicola* species. In contrast, the evolution of *Corrosella* species in headwaters is probably driven by non-ecological processes. Therefore, our results showed the evolutionary significance of elevation along the river continuum and add an example to the growing body of evidence from other freshwater groups that ecological opportunity plays a key role in species diversification. We encourage future macroevolutionary studies on diversification processes in non-marine aquatic organisms for a more comprehensive understanding of patterns and causes of diversification across taxa and habitat-types.

## Additional files

Additional file 1: **Table S1.** Species names, locality data, locality codes and GenBank accession numbers for the taxa studied. **Table S3.** Abbreviations of the morphological characters depicted in Additional file 3: Table S2. **Figure S2.** Comparison of macroevolutionary models. Boxplots showing the relative fit (ΔAIC) of quantitative state speciation and extinction (QuaSSE) models estimating the evolution of temperature preference and its potential influence on speciation rate along 100 random post-burn-in trees of Pseudamnicolinae for (a) mean annual temperature, (b) mean temperature of the warmest season, and (c) mean temperature of the coldest season. Each set of temperature included a standard deviation (SD) of 10, 20, 30%. Dots display the relative fit for the MCC tree. **Tables S4, S5.** Models testing the relationship of speciation rates with both environmental temperature and elevation, respectively. We fitted quantitative state speciation and extinction (QuaSSE) models with genus specific or independent coefficients for rates of speciation (λ), change of speciation rate with elevation (λ_Elv_) and environmental temperature (λ_Tem_), and rates of elevational and environmental temperature evolution (σ^2^). Subscripts P and C denote the two genera *Pseudamnicola* and *Corrosella*, respectively. Empty cells indicate identical coefficients of the respective parameter for both genera. Not available (NA) parameters were not included in the respective model. For each model, the first row indicates the coefficients and model fit of the MCC tree. Brackets in the second row include the average and standard deviation of these coefficients as well as the model fit based on 100 random post-burn-in trees. **Table S6.** Testing mode and rates of morphological divergence of the two sister-genera *Pseudamnicola* and *Corrosella* along the MCC. The Brownian Motion (BM) model includes the rate of morphological divergence (σ^2^) and the morphological optimum (θ). We used AIC-based model comparison to test additional variants of this model that allowed for genus and/or trait specific coefficients. Empty cells indicate identical coefficients for both trait axes or both genera, respectively. For each tested model, the first row indicates the coefficients and model fit of the MCC tree and the brackets in the second row include the average and standard deviation of these coefficients as well as the model fit based on 100 random post-burn-in trees. Because of the relatively small number of species, we could not fit more complex models with genus- and trait specific parameters or selection. (PDF 625 kb)

Additional file 2: **Figure S1.** Phylogenetic relationships of *Corrosella* and *Pseudamnicola* species based on a Bayesian inference of the combined COI, 16S, and 28S datasets. Bayesian posterior probabilities are indicated with black dots when < 0.9. Bars on the right denote species assignments. For locality codes see Additional file 1: Table S1. (PDF 2138 kb)

Additional file 3: **Table S2.** Morphological dataset. Abbreviations explained in Additional file 1: Table S3. (TXT 5 kb)
