# Supplementary material for: Ecological opportunity may facilitate diversification in Palearctic freshwater organisms: a case study on hydrobiid gastropods
Source: BMC Evol Biol. 2018 Apr 19;18:55. doi: 10.1186/s12862-018-1169-2 (PMC5907725; doi:10.1186/s12862-018-1169-2)
Supplement: Supplementary file 1 — Table S1. Species names, locality data, locality codes and GenBank accession numbers for the taxa studied. Table S3. Abbreviations of the morphological characters depicted in Additional file 3: Table S2. Figure S2. Comparison of macroevolutionary models. Boxplots showing the relative fit (ΔAIC) of quantitative state speciation and extinction (QuaSSE) models estimating the evolution of temperature preference and its potential influence on speciation rate along 100 random post-burn-in trees of Pseudamnicolinae for (a) mean annual temperature, (b) mean temperature of the warmest season, and (c) mean temperature of the coldest season. Each set of temperature included a standard deviation (SD) of 10, 20, 30%. Dots display the relative fit for the MCC tree. Tables S4, S5. Models testing the relationship of speciation rates with both environmental temperature and elevation, respectively. We fitted quantitative state speciation and extinction (QuaSSE) models with genus specific or independent coefficients for rates of speciation (λ), change of speciation rate with elevation (λElv) and environmental temperature (λTem), and rates of elevational and environmental temperature evolution (σ2). Subscripts P and C denote the two genera Pseudamnicola and Corrosella, respectively. Empty cells indicate identical coefficients of the respective parameter for both genera. Not available (NA) parameters were not included in the respective model. For each model, the first row indicates the coefficients and model fit of the MCC tree. Brackets in the second row include the average and standard deviation of these coefficients as well as the model fit based on 100 random post-burn-in trees. Table S6. Testing mode and rates of morphological divergence of the two sister-genera Pseudamnicola and Corrosella along the MCC. The Brownian Motion (BM) model includes the rate of morphological divergence (σ2) and the morphological optimum (θ). We used AIC-based model comparison to test additional variants [file 12862_2018_1169_MOESM1_ESM.pdf]

Additional file 1: Table S1. Species names, locality data, locality codes, number of sequenced specimens, and GenBank accession numbers for the taxa studied.

Species	Locality	Code	# specimens	GenBank accession # COI/16S/28S
Outgroup				
Mercuria emiliana (Paladilhe, 1869)	Ullal Baltasar, Amposta, Tarragona, Spain (40.670868, 0.586862)		1	JX081888 Delicado et al., 2013<br>JX081990 Delicado et al., 2013<br>JX081779 Delicado et al., 2013
Diegus gasulli (Boeters, 1981)	Retamar Rambla, Almería, Spain		1	KF060741 Delicado et al., 2014<br>KF060804 Delicado et al., 2014<br>KF060867 Delicado et al., 2014
Ingroup				
Corrosella andalusica (Delicado, Machordom & Ramos, 2012)	La Salud Spring, Albanchez de Magina, Córdoba, Spain (37.780164, -3.468784)	Sal	1	JF312223 Delicado et al., 2012<br>JX081893 Delicado et al., 2013<br>JX081682 Delicado et al., 2013
	Eduardo Spring, Alcaucín, Málaga, Spain (36.916802, -4.091905)	Edu	1	JX081805 Delicado et al., 2013<br>JX081894 Delicado et al., 2013<br>JX081683 Delicado et al., 2013
	El Piojo Spring, Almedinilla, Córdoba, Spain (37.433331, -4.084560)	Pio	1	JX081806 Delicado et al., 2013<br>JX081895 Delicado et al., 2013<br>JX081684 Delicado et al., 2013
C. astierii (Dupuy, 1851)	Source de Argens, Brue-Aurillac, Var, France (43.518674, 5.907080)	Arg	3	JQ067672-4 Delicado & Ramos, 2012<br>JX081890-2 Delicado et al., 2013<br>JX081679-81 Delicado et al., 2013
Corrosella sp. 1	Spring in Jatar, Granada, Spain (36.939469, -3.919170)	Jat	3	JX081807-9 Delicado et al., 2013<br>JX081896-8 Delicado et al., 2013<br>JX081685-7 Delicado et al., 2013
C. bareai (Delicado, Machordom & Ramos, 2012)	Spring at Ermita de las Santas, Collados de la Sagra, Granada, Spain (37.433331, -4.086219)	San	4	JF312225-6 JX081810-11 Delicado et al., 2012; 2013<br>JX081899-902 Delicado et al., 2013<br>JX081688-91 Delicado et al., 2013

Species	Locality	Code	# specimens	GenBank accession # COI/16S/28S
	Siete Fuentes Spring, Cuenca, Jaén, Spain (37.739786, -2.971598)	Sie	1	JX081816 Delicado et al., 2013<br>JX081907 Delicado et al., 2013<br>JX081696 Delicado et al., 2013
	El Laude Spring, Castril, Granada, Spain (37.832692, -2.805230)	Lau	1	JX081813 Delicado et al., 2013<br>JX081904 Delicado et al., 2013<br>JX081693 Delicado et al., 2013
	Fuente Nuevas Spring, Castril, Granada, Spain (37.78663, -2.869028)	Nue	2	JX081814-5 Delicado et al., 2013<br>JX081905-6 Delicado et al., 2013<br>JX081694-5 Delicado et al., 2013
	Aguerillo Spring, Castril, Granada, Spain (37.829579, -2.806292)	Agu	1	JX081812 Delicado et al., 2013<br>JX081903 Delicado et al., 2013<br>JX081692 Delicado et al., 2013
	La Plata Spring, Riopar, Albacete, Spain (38.487450, -2.324452)	Pla	3	JX081817-9 Delicado et al., 2013<br>JX081908-10 Delicado et al., 2013<br>JX081697-9 Delicado et al., 2013
C. falkneri Boeters, 1970	La Armada Spring, Orce, Granada, Spain (37.731357, -2.476429)	Arm	3	JF312224 JX081820-1 Delicado et al., 2012; 2013<br>JX081911-3 Delicado et al., 2013<br>JX081700-2 Delicado et al., 2013
	Palo Spring, Orce, Granada, Spain (37.726336, -2.487574)	Pal	1	JX081822 Delicado et al., 2013<br>JX081914 Delicado et al., 2013<br>JX081703 Delicado et al., 2013
	Spring in Castril, Granada, Spain (37.819122, -2.768609)	Cas	3	JX081823-5 Delicado et al., 2013<br>JX081915-7 Delicado et al., 2013<br>JX081704-6 Delicado et al., 2013
	La Erra Spring, La Dehesa, Albacete, Spain (38.327325, -2.172965)	Deh	2	JX081826-7 Delicado et al., 2013<br>JX081918-9 Delicado et al., 2013<br>JX081707-8 Delicado et al., 2013
C. hauffei (Delicado & Ramos, 2012)	Los Nogales Spring, Benafer, Castellón, Spain (39.930003, -0.573629)	Nog	3	JQ067675-7 Delicado & Ramos, 2012<br>JX081920-2 Delicado et al., 2013<br>JX081709-11 Delicado et al., 2013

Species	Locality	Code	# specimens	GenBank accession # COI/16S/28S
	Curso Spring, Navajas, Castellón, Spain (39.873780, -0.501126)	Cur	3	JX081828-30 Delicado et al., 2013<br>JX081923-5 Delicado et al., 2013<br>JX081712-4 Delicado et al., 2013
	San Miguel Spring, Viver, Castellón, Spain (39.925455, -0.611904)	SMi	2	JX081831-2 Delicado et al., 2013<br>JX081926-7 Delicado et al., 2013<br>JX081715-6 Delicado et al., 2013
C. hinzi (Boeters, 1986)	Prado Spring, Caminreal, Teruel, Spain (40.845159, -1.334301)	Cam	2	JX081839-40 Delicado et al., 2013<br>JX081934-5 Delicado et al., 2013<br>JX081723-4 Delicado et al., 2013
	Spring of river park in Calamocha, Teruel, Spain (40.926091, -1.298874)	Cal	1	JX081838 Delicado et al., 2013<br>JX081933 Delicado et al., 2013<br>JX081722 Delicado et al., 2013
	Cazuelas Spring, Borja, Zaragoza, Spain (41.822531, -1.550223)	Caz	2	JX081836-7 Delicado et al., 2013<br>JX081931-2 Delicado et al., 2013<br>JX081720-1 Delicado et al., 2013
	Vargas Balsa, Borja, Zaragoza, Spain (41.825353, -1.552491)	Var	3	JX081833-5 Delicado et al., 2013<br>JX081928-30 Delicado et al., 2013<br>JX081717-9 Delicado et al., 2013
C. iruritai (Delicado, Machordom & Ramos, 2012)	Don Pedro Spring, Loja, Granada, Spain (37.177648, -4.133082)	DPe	3	JX081841 JF312221-2 Delicado et al., 2012; 2013<br>JX081936-8 Delicado et al., 2013<br>JX081725-7 Delicado et al., 2013
C. luisi (Boeters, 1984)	La Gitana Spring, La Peza, Granada, Spain (37.268965, -3.293065)	Git	3	JF312220 JX081842-3 Delicado et al., 2012; 2013<br>JX081939-41 Delicado et al., 2013<br>JX081728-30 Delicado et al., 2013
	Polvorista Stream, Quentar, Granada, Spain (37.253212, -3.393349)	Pol	1	JX081844 Delicado et al., 2013<br>JX081942 Delicado et al., 2013<br>JX081731 Delicado et al., 2013
	La Teja Spring, Sierra de Huetor, Granada, Spain (37.266359, -3.508451)	Tej	2	JX081845-6 Delicado et al., 2013<br>JX081943-4 Delicado et al., 2013<br>JX081732-3 Delicado et al., 2013

Species	Locality	Code	# specimens	GenBank accession # COI/16S/28S
C. manueli (Delicado, Machordom & Ramos, 2012)	La Garganta Stream, Nava de San Pedro, Jaen, Spain (37.899453, -2.895526)	Gar	2	JF312227-8 Delicado et al., 2012<br>JX081945-6 Delicado et al., 2013<br>JX081734-5 Delicado et al., 2013
	San Isidro Ditch, Cazorla, Jaen, Spain (37.912344, -3.003362)	Isi	2	JX081850-1 Delicado et al., 2013<br>JX081950-1 Delicado et al., 2013<br>JX081739-40 Delicado et al., 2013
	El Céfano Spring, La Iruela, Jaen, Spain (37.929755, -2.975872)	Cef	1	JX081849 Delicado et al., 2013<br>JX081949 Delicado et al., 2013<br>JX081738 Delicado et al., 2013
	El Valle Stream, La Iruela, Jaen, Spain (37.919780, -2.955886)	Val	2	JX081847-8 Delicado et al., 2013<br>JX081947-8 Delicado et al., 2013<br>JX081736-7 Delicado et al., 2013
C. marisolae (Delicado, Machordom & Ramos, 2012)	Pilar del Mono Spring, Durcal, Granada, Spain (37.003064, -3.572243)	PMo	2	JF312218-9 Delicado et al., 2012<br>JX081952-3 Delicado et al., 2013<br>JX081741-2 Delicado et al., 2013
	Spring in Padul, Granada, Spain (37.026073, -3.625610)	Pad	2	JX081852-3 Delicado et al., 2013<br>JX081954-5 Delicado et al., 2013<br>JX081743-4 Delicado et al., 2013
C. navasiana (Fagot, 1907)	Spring in Peralejos de las truchas, Guadalajara, Spain (40.615672, -1.970116)	Per	1	JX081867 Delicado et al., 2013<br>JX081969 Delicado et al., 2013<br>JX081758 Delicado et al., 2013
	Maria Spring, Ontígola, Toledo, Spain (39.997856, -3.577435)	Mar	3	JX081880-2 Delicado et al., 2013<br>JX081982-4 Delicado et al., 2013<br>JX081771-3 Delicado et al., 2013
	Ditch in Borox, Toledo, Spain (40.057145, -3.738823)	Box	3	JX081883-5 Delicado et al., 2013<br>JX081985-7 Delicado et al., 2013<br>JX081774-6 Delicado et al., 2013
	Stream in Tubilleja, Burgos, Spain (42.853529, -3.714851)	Tub	1	JX081878 Delicado et al., 2013<br>JX081980 Delicado et al., 2013<br>JX081769 Delicado et al., 2013

Species	Locality	Code	# specimens	GenBank accession # COI/16S/28S
	Valdemenez Stream, Sedano, Burgos, Spain (42.716257, -3.800713)	Vld	1	JX081877 Delicado et al., 2013<br>JX081979 Delicado et al., 2013<br>JX081768 Delicado et al., 2013
	La Toba Spring, Tubilla del agua, Burgos, Spain (42.726813, -3.752895)	Tob	1	JX081876 Delicado et al., 2013<br>JX081978 Delicado et al., 2013<br>JX081767 Delicado et al., 2013
	Pozo Azul, Covanera, Burgos, Spain (42.739591, -3.797638)	Poz	3	JX081873-5 Delicado et al., 2013<br>JX081975-7 Delicado et al., 2013<br>JX081764-6 Delicado et al., 2013
	Lago del Espejo, Nuevalos, Zaragoza, Spain (41.197493, -1.788075)	Esp	1	JX081864 Delicado et al., 2013<br>JX081966 Delicado et al., 2013<br>JX081755 Delicado et al., 2013
	Tia Perra Spring, El Hosquillo, Cuenca, Spain (40.370856, -2.005384)	Tia	2	JX081871-2 Delicado et al., 2013<br>JX081973-4 Delicado et al., 2013<br>JX081762-3 Delicado et al., 2013
	Spring in Arbujuelo, Soria, Spain (41.136085, -2.379054)	Arb	1	JX081866 Delicado et al., 2013<br>JX081968 Delicado et al., 2013<br>JX081757 Delicado et al., 2013
	Ojos de Cimballa Wetland, Zaragoza, Spain (41.090624, -1.768418)	Cim	1	JX081862 Delicado et al., 2013<br>JX081964 Delicado et al., 2013<br>JX081753 Delicado et al., 2013
	Tinte Spring, Medinaceli, Soria, Spain (41.159197, -2.427141)	Tin	1	JX081865 Delicado et al., 2013<br>JX081967 Delicado et al., 2013<br>JX081756 Delicado et al., 2013
	Dulce River, Cabrera, Guadalajara, Spain (41.007661, -2.676663)	Dul	1	JX081870 Delicado et al., 2013<br>JX081972 Delicado et al., 2013<br>JX081761 Delicado et al., 2013
	Stream in Valtubilla, Sedano, Burgos, Spain (42.726813, -3.752895)	Vat	1	JX081879 Delicado et al., 2013<br>JX081981 Delicado et al., 2013<br>JX081770 Delicado et al., 2013

Species	Locality	Code	# specimens	GenBank accession # COI/16S/28S
	Spring in Mesones, Zaragoza, Spain (41.551747, -1.536194)	Mes	1	JX081863 Delicado et al., 2013<br>JX081965 Delicado et al., 2013<br>JX081754 Delicado et al., 2013
	Source of Bornova river, Guadalajara, Spain (41.257243, -3.075080)	Bor	1	JX081869 Delicado et al., 2013<br>JX081971 Delicado et al., 2013<br>JX081760 Delicado et al., 2013
	Stream near Canalejas Spring, Somolinos, Guadalajara, Spain (41.257743, -3.070976)	Can	1	JX081868 Delicado et al., 2013<br>JX081970 Delicado et al., 2013<br>JX081759 Delicado et al., 2013
	Fonnueva Spring, Bulbuente, Zaragoza, Spain (41.818282, -1.387294)	Fon	8	JX081854-61 Delicado et al., 2013<br>JX081956-63 Delicado et al., 2013<br>JX081745-52 Delicado et al., 2013
Pseudamnicola artanensis Altaba, 2007	Spring near Betlem Hermitage, Arta, Majorca Island, Spain (39.735990, 3.315565)	Bet	3	KF060721-3 Delicado et al., 2014<br>KF060784-6 Delicado et al., 2014<br>KF060847-9 Delicado et al., 2014
P. beckmanni Glöer & Zettler, 2007	El Rentador Spring, Deya, Majorca Island, Spain (39.746359, 2.649290)	Ren	3	KF060724-6 Delicado et al., 2014<br>KF060787-9 Delicado et al., 2014<br>KF060850-2 Delicado et al., 2014
	Spring in Valldemossa, Majorca Island, Spain (39.710083, 2.625862)	Vad	2	KF060727-8 Delicado et al., 2014<br>KF060790-1 Delicado et al., 2014<br>KF060853-4 Delicado et al., 2014
	Spring in Randa, Majorca Island, Spain (39.542504, 2.905000)	Ran	2	KF060729-30 Delicado et al., 2014<br>KF060792-3 Delicado et al., 2014<br>KF060855-6 Delicado et al., 2014
	Stream at the gardens of the Palace La Granja, Esporles, Majorca Island, Spain (39.668327, 2.558454)	LGr	5	KF060731-5 Delicado et al., 2014<br>KF060794-8 Delicado et al., 2014<br>KF060857-61 Delicado et al., 2014
	Jardins de Alfabia, Majorca Island, Spain (39.66700, 2.692520)	Alf	2	KM668755-6 Delicado et al., 2015<br>KM668795-6 Delicado et al., 2015<br>KM668835-6 Delicado et al., 2015

Species	Locality	Code	# specimens	GenBank accession # COI/16S/28S
P. granjaensis Glöer & Zettler, 2007	Fountain near the exit of the Palace La Granja, Esporles, Majorca Island, Spain (39.668796, 2.558626)	Gra	3	KF060744-6 Delicado et al., 2014<br>KF060807-9 Delicado et al., 2014<br>KF060870-2 Delicado et al., 2014
P. meloussensis Altaba, 2007	Spring at Macarella Creek, Minorca Island, Spain (39.939511, 3.937255)	Mac	10	KF060747-56 Delicado et al., 2014<br>KF060810-9 Delicado et al., 2014<br>KF060873-82 Delicado et al., 2014
	Font de la Reina, Son Saura, Minorca Island, Spain (39.934879, 3.899810)	FRe	5	KF060757-61 Delicado et al., 2014<br>KF060820-4 Delicado et al., 2014<br>KF060883-7 Delicado et al., 2014
	Stream at Barranco de Son Boter, Minorca Island, Spain (39.919998, 4.042499)	SBo	5	KF060762-6 Delicado et al., 2014<br>KF060825-9 Delicado et al., 2014<br>KF060888-92 Delicado et al., 2014
	Stream at Barranco de Sen Penyes, Minorca Island, Spain (39.853482, 4.167928)	SPe	5	KF060767-71 Delicado et al., 2014<br>KF060830-4 Delicado et al., 2014<br>KF060893-7 Delicado et al., 2014
P. negropontina (Clessin, 1878)	Marmari, South Evia Island, Greece (38.039300, 24.327500)	Mrm	2	KM668770-1 Delicado et al., 2015<br>KM668810-1 Delicado et al., 2015<br>KM668850-1 Delicado et al., 2015
P. subproducta (Paladilhe, 1869)	Font Estramar near Salses le Chateau, France (42.858900, 2.957317)	Est	1	KF060772 Delicado et al., 2014<br>KF060835 Delicado et al., 2014<br>KF060898 Delicado et al., 2014
	Les Borges del Camp, Tarragona, Spain (41.171665, 1.019722)	LBo	2	KF060773-4 Delicado et al., 2014<br>KF060836-7 Delicado et al., 2014<br>KF060899-900 Delicado et al., 2014
	Prado de Cifuentes Spring, Calatayud, Zaragoza, Spain (41.333740, -1.660406)	Cif	3	KF060775-7 Delicado et al., 2014<br>KF060838-40 Delicado et al., 2014<br>KF060901-3 Delicado et al., 2014
	Ontígola Lake, Madrid, Spain (40.018323, -3.601157)	Ont	3	KF060778-80 Delicado et al., 2014<br>KF060841-3 Delicado et al., 2014<br>KF060904-6 Delicado et al., 2014

Species	Locality	Code	# specimens	GenBank accession # COI/16S/28S
	Flores Spring, Requena, Valencia, Spain (39.489554, -1.126306)	Flo	3	KF060781-3 Delicado et al., 2014<br>KF060844-6 Delicado et al., 2014<br>KF060907-9 Delicado et al., 2014
	Chalissane St. Chamas, Etang de Berre, Bouches du Rhone, France (37.993000, 13.289000)	Cha	1	KM668774 Delicado et al., 2015<br>KM668814 Delicado et al., 2015<br>KM668854 Delicado et al., 2015
	Ullal Baltasar, Amposta, Tarragona, Spain (40.670864, 0.586862)	Ull	2	JX081886-7 Delicado et al., 2013<br>JX081988-9 Delicado et al., 2013<br>JX081777-8 Delicado et al., 2013
P. conovula (Frauenfeld, 1863)	Spring near Napoli, Italy (40.845643, 14.267952)	Nap	1	KM668776 Delicado et al., 2015<br>KM668816 Delicado et al., 2015<br>KM668856 Delicado et al., 2015
	Giammatteo Creek, Lecce, Italy (44.943580, 12.021270)	Gia	2	KM668777-8 Delicado et al., 2015<br>KM668817-8 Delicado et al., 2015<br>KM668857-8 Delicado et al., 2015
	Spring near the church of S. Maria della Foce, Napoli, Campania, Italy (40.83369, 14.594070)	Foc	1	MG697213 Present study<br>MG697198 Present study<br>MG697205 Present study
	T. Spartifavo, 250 NW of the Pietra d. Pertusillo Lake, Viggiano, Italy (40.295863, 15.917747)	Spa	1	MG697214 Present study<br>MG697199 Present study<br>MG697206 Present study
	Kastelanci Spring, Rtina, Croatia (44.272500, 15.312490)	Kas	1	MG697215 Present study<br>MG697200 Present study<br>MG697207 Present study
P. lucensis (Issel, 1866)	Spring at the road to Baratti, near the Necropoli Etrusca, Grosseto, Tuscany, Italy (42.991880, 10.496570)	Tus	1	KM668775 Delicado et al., 2015<br>KM668815 Delicado et al., 2015<br>KM668855 Delicado et al., 2015
	Bagni Caldi, Bagni di Lucca, Lucca Tuscany, Italy (44.008340, 10.571793)	BCa	1	AF367651 Wilke et al., 2001<br>AF478394 Wilke et al., 2001<br>MG697212 Present study

Species	Locality	Code	# specimens	GenBank accession # COI/16S/28S
	Spring near Casaccina, Santa Luce, Pisa, Tuscany, Italy (42.991883, 10.496570)	SLu	1	MG697216 Present study<br>MG697201 Present study<br>MG697208 Present study
P. calamensis Glöer, Bouzid & Boeters, 2010	Grotta Fontana, Cossoine, Sardinia, Italy (40.424019, 8.714722)	GFo	1	KM668786 Delicado et al., 2015<br>KM668826 Delicado et al., 2015<br>KM668866 Delicado et al., 2015
	Grotta Sorigalza, Cossoine, Sardinia, Italy (40.424019, 8.714722)	GSo	2	KM668787-8 Delicado et al., 2015<br>KM668827-8 Delicado et al., 2015<br>KM668867-8 Delicado et al., 2015
P. moussoni (Calcara, 1841)	Spring near the trough of Balateddi, Portella road, Sicily, Italy (37.953390, 13.304043)	Bal	2	KM668763-4 Delicado et al., 2015<br>KM668803-4 Delicado et al., 2015<br>KM668843-4 Delicado et al., 2015
	Spring at Menfi-Sciacca Road, Sicily, Italy (37.517240, 13.092980)	MSi	2	KM668765-6 Delicado et al., 2015<br>KM668805-6 Delicado et al., 2015<br>KM668845-6 Delicado et al., 2015
	Spring near the Signalmans, Sicily, Italy (38.016420, 14.122790)	Sig	2	KM668767-8 Delicado et al., 2015<br>KM668807-8 Delicado et al., 2015<br>KM668847-8 Delicado et al., 2015
Pseudamnicola sp. 1	Spring below the Fontana Carpina, M. Argentario, Tuscany, Italy (42.427532, 11.098644)	MAr	1	MG697217 Present study<br>MG697202 Present study<br>MG697209 Present study
Pseudamnicola sp. 2	Borkane Ditch, Tunisia (37.105293, 9.856347)	Brk	1	KM668779 Delicado et al., 2015<br>KM668819 Delicado et al., 2015<br>KM668859 Delicado et al., 2015
	Ditch towards the north of Ichkeul, Tunisia (37.205506, 9.667662)	Ich	2	KM668780-1 Delicado et al., 2015<br>KM668820-1 Delicado et al., 2015<br>KM668860-1 Delicado et al., 2015
	Stream near Bizerte, Tunisia (37.242626, 9.718454)	Biz	4	KM668782-5 Delicado et al., 2015<br>KM668822-5 Delicado et al., 2015<br>KM668862-5 Delicado et al., 2015

Species	Locality	Code	# specimens	GenBank accession # COI/16S/28S
Pseudamnicola sp. 3	Spring in Via Marrucella, Capri Island, Italy (40.549210, 14.232330)	Cap	2	KM668757-8 Delicado et al., 2015<br>KM668797-8 Delicado et al., 2015<br>KM668837-8 Delicado et al., 2015
	Grotta Fontana, Cossoine, Sardinia, Italy (40.424019, 8.714722)	GFo	1	KM668759 Delicado et al., 2015<br>KM668799 Delicado et al., 2015<br>KM668839 Delicado et al., 2015
	Spring on the left bank of T. Biedano, 100m upstream of the bridge under Blera, Latium, Italy (42.268023, 12.025564)	Lat	2	KM668760-1 Delicado et al., 2015<br>KM668800-1 Delicado et al., 2015<br>KM668840-1 Delicado et al., 2015
	Stream near Sperlonga, Italy (41.257980, 13.434800)	Spe	1	KM668762 Delicado et al., 2015<br>KM668802 Delicado et al., 2015<br>KM668842 Delicado et al., 2015
Pseudamnicola sp. 4	Borkane Ditch, Tunisia (37.105293, 9.856347)	Brk	3	KM668789-91 Delicado et al., 2015<br>KM668829-31 Delicado et al., 2015<br>KM668869-71 Delicado et al., 2015
	Chafrou River, Tunisia (36.833566, 9.948237)	Chf	1	KM668792 Delicado et al., 2015<br>KM668832 Delicado et al., 2015<br>KM668872 Delicado et al., 2015
	Stream near Chaouat, Tunisia (36.880857, 9.938890)	Cho	2	KM668793-4 Delicado et al., 2015<br>KM668833-4 Delicado et al., 2015<br>KM668873-4 Delicado et al., 2015
Pseudamnicola sp. 5	Fiumefreddo, Sicily, Italy (37.785130, 15.227140)	Fiu	2	KM668772-3 Delicado et al., 2015<br>KM668812-3 Delicado et al., 2015<br>KM668852-3 Delicado et al., 2015
Pseudamnicola sp. 6	Spring between Valle Bucceri and Valle Ficarella, Isle of Marettimo, Favignana, Trapani, Sicily, Italy (37.973990, 12.062770)	Tra	1	KM668769 Delicado et al., 2015<br>KM668809 Delicado et al., 2015<br>KM668849 Delicado et al., 2015
Pseudamnicola sp. 7	Bau sa Mela River, Nurallao, Sardinia, Italy (39.839961, 9.105191)	Bau	1	MG697218 Present study<br>MG697203 Present study<br>MG697210 Present study

Species	Locality	Code	# specimens	GenBank accession # COI/16S/28S
Pseudamnicola sp. 8	Spring near the pyrite-mine, Pianizzoli, Grosseto, Tuscany, Italy (43.038839, 10.964200)	Pia	1	MG697219 Present study<br>MG697204 Present study<br>MG697211 Present study

Table S3. Abbreviations of the morphological characters depicted in Table S2.

Abbreviation	Morphological character
SL	Shell length
SW	Shell width
LBW	Length body whorl
NSW	Number of spire whorls
ProL	Protoconch length
NSWPro	Number of spire whorls of protoconch
Promicro	Protoconch microsculpture
AL	Aperture length
AW	Aperture width
Umbilicus	Umbilicus size
RadL	Length of the radular ribbon
Nrows	Number of rows of teeth
CToothCusps	Number of lateral cusps on the central tooth
LToothCusps	Number of lateral cusps on the lateral tooth
IMTeethCusps	Number of cusps on the inner marginal teeth
OMTeethCusps	Number of cusps on the outer marginal teeth
Gill	Number of gill filaments
BursaShape	Shape of the bursa copulatrix
BuductL	Length of the bursal duct
BudL/BuL	Length of the bursal duct / length of the bursa copulatrix
Srshape	Shape of the seminal receptacle
SRL	Length of the seminal receptacle
Srduct	Length of the seminal receptacle duct
PL/Head	Length of the penis / length of the head
ProstateL	Length of the prostate gland
RPG	Nervous system RPG ratio

Figure S2. Comparison of macroevolutionary models. Boxplots showing the relative fit (ΔAIC) of quantitative state speciation and extinction (QuaSSE) models estimating the evolution of temperature preference and its potential influence on speciation rate along 100 random post-burn-in trees of Pseudamnicolinae for (a) mean annual temperature, (b) mean temperature of the warmest season, and (c) mean temperature of the coldest season. Each set of temperature included a standard deviation (SD) of 10, 20, 30%. Dots display the relative fit for the MCC tree.

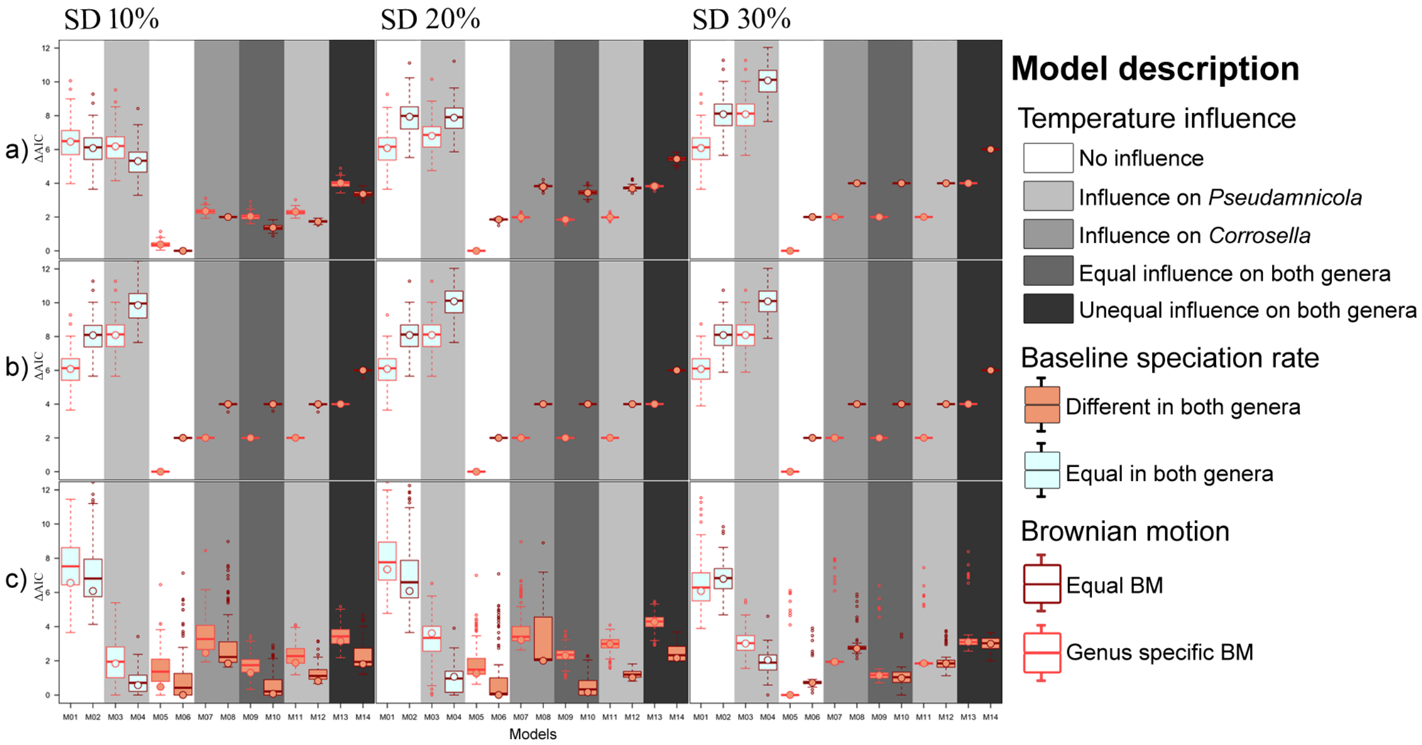

Table S4: Models testing the relationship between environmental temperature and speciation rate. We fitted quantitative state speciation and extinction (QuaSSE) models with genus specific or independent coefficients for rates of speciation (λ), change of speciation rate with temperature (λ_{Tem}), and rates of temperature evolution (σ^2). Subscript P and C denote the two genera *Pseudamnicola* and *Corrosella*, respectively. Empty cells indicate identical coefficients of the respective parameter for both genera. Not available (NA) parameters were not included in the respective model. For each model, the first row indicates the coefficients and model fit of the MCC tree and the brackets in the second row include the average and standard deviation of these coefficients as well as the model fit based on 100 random post-burn-in trees.

Model	λ_P	$\lambda_{P\ Tem}$	σ^2_P	λ_C	$\lambda_{C\ Tem}$	σ^2_C	ΔAIC	Model rank
1	5.20	NA	1.23		NA		6.46	14
	[5.13±0.43]		[1.22±0.06]				[6.46±1.17]	[13.91±0.32]
2	5.20	NA	3.58		NA	0.00	6.08	12
	[5.13±0.03]		[3.58±0.07]			[0.00±0.00]	[6.08±1.06]	[12.22±0.46]
3	0.00	0.36	1.38				6.18	13
	[0.00±0.00]	[0.35±0.03]	[1.38±0.07]				[6.19±1.03]	[12.84±0.55]
4	0.00	0.36	3.85			0.00	5.30	11
	[0.00±0.00]	[0.36±0.03]	[3.86±0.26]			[0.00±0.00]	[5.32±0.92]	[10.94±0.28]
5	7.84	NA	1.23	2.96	NA		0.38	2
	[7.73±0.76]		[1.22±0.06]	[2.93±0.29]			[0.38±0.18]	[2.00±0.00]
6	7.84	NA	3.58	2.96	NA	0.00	0.00	1
	[7.73±0.76]		[3.58±0.23]	[2.93±0.29]		[0.00±0.00]		[1.00±0.00]
7	7.84	NA	1.21	6.98	-0.29		2.35	8
	[7.73±0.76]		[1.20±0.07]	[7.00±4.12]	[-0.29±0.28]		[2.33±0.20]	[7.72±0.47]
8	7.84	NA	3.58	2.96	0.00	0.00	2.00	5
	[7.73±0.76]		[3.58±0.23]	[2.84±0.50]	[0.01±0.03]	[0.00±0.00]	[2.00±0.00]	[5.51±0.66]
9	0.00	0.50	1.23	2.96	NA		2.04	6
	[0.04±0.36]	[0.50±0.06]	[1.22±0.06]	[2.93±0.29]			[2.04±0.21]	[5.58±0.55]

10	0.00	0.50	3.58	2.96	NA	0.00	1.37	3
	[0.00±0.00]	[0.50±0.05]	[3.59±0.26]	[2.93±0.29]		[0.00±0.00]	[1.37±0.18]	[3.00±0.00]
11	4.45	0.22	1.23	0.00			2.32	7
	[4.48±1.09]	[0.21±0.07]	[1.23±0.06]	[0.09±0.86]			[2.29±0.18]	[7.19±0.51]
12	4.37	0.22	3.58	0.00		0.00	1.73	4
	[4.31±0.52]	[0.22±0.02]	[3.58±0.24]	[0.00±0.00]		[0.00±0.00]	[1.73±0.08]	[4.00±0.00]
13	0.00	0.50	1.21	6.29	-0.24		4.02	10
	[0.82±8.22]	[0.45±0.52]	[1.22±0.12]	[6.25±3.86]	[-0.24±0.26]		[4.00±0.23]	[10.07±0.41]
14	0.00	0.50	3.58	5.58	-0.19	0.00	3.37	9
	[0.00±0.00]	[0.50±0.05]	[3.59±0.26]	[4.01±1.23]	[-0.08±0.08]	[0.00±0.00]	[3.37±0.18]	[9.02±0.14]

Table S5: Models testing the relationship between elevation and speciation rate. We fitted quantitative state speciation and extinction (QuaSSE) models with genus specific or independent coefficients for rates of speciation (λ), change of speciation rate with elevation (λ_{Elv}), and rates of elevational evolution (σ^2). Subscript P and C denote the two genera *Pseudamnicola* and *Corrosella*, respectively. Empty cells indicate identical coefficients of the respective parameter for both genera. Not available (NA) parameters were not included in the respective model. For each model, the first row indicates the coefficients and model fit of the MCC tree and the brackets in the second row include the average and standard deviation of these coefficients as well as the model fit based on 100 random post-burn-in trees.

Model	λ_P	$\lambda_{P\ Elv}$	σ^2_P	λ_C	$\lambda_{C\ Elv}$	σ^2_C	ΔAIC	Model rank
1	5.20	NA	0.07		NA		8.76	13
	[5.17±0.41]		[0.07±0.01]				[8.82±1.83]	[12.96±0.24]
2	5.20	NA	0.10		NA	0.04	10.21	14
	[5.17±0.41]		[0.10±0.01]			[0.04±0.01]	[10.23±1.74]	[14.00±0.00]
3	12.21	-9.97	0.08				2.47	5
	[12.35±1.67]	[-10.13±1.60]	[0.08±0.01]				[2.40±0.87]	[4.93±1.20]
4	13.22	-11.17	0.12			0.03	2.09	4
	[13.30±1.73]	[-11.27±1.66]	[0.13±0.02]			[0.03±0.01]	[1.98±0.68]	[3.89±1.10]
5	7.84	NA	0.07	2.96	NA		2.68	6
	[7.76±0.69]		[0.07±0.01]	[2.96±0.26]			[2.88±1.35]	[5.84±1.87]
6	7.84	NA	0.10	2.96	NA	0.04	4.13	9
	[7.76±0.69]		[0.10±0.01]	[2.96±0.26]		[0.04±0.01]	[4.29±1.34]	[8.96±1.15]
7	7.84	NA	0.07	0.00	3.39		4.27	10
	[7.76±0.69]		[0.07±0.01]	[0.01±0.08]	[3.38±0.35]		[4.46±1.30]	[9.46±1.34]
8	7.84	NA	0.09	0.01	3.35	0.05	5.84	12
	[7.76±0.69]		[0.10±0.01]	[0.03±0.16]	[3.32±0.39]	[0.05±0.01]	[5.98±1.30]	[11.79±0.46]
9	19.99	-25.52	0.07	2.96	NA		0	1
	[18.88±5.55]	[-22.76±12.95]	[0.07±0.02]	[2.96±0.26]				[1.30±1.30]

10	18.77	-22.55	0.10	2.96	NA	0.04	0.93	2
	[17.91±5.30]	[-20.39±11.46]	[0.10±0.01]	[2.96±0.26]		[0.04±0.02]	[1.14±1.36]	[2.66±2.33]
11	13.41	-12.66	0.08	14.88			4.35	11
	[10.24±4.00]	[-5.34±8.40]	[0.08±0.01]	[8.07±7.89]			[4.27±1.07]	[9.66±1.25]
12	14.63	-14.50	0.12	16.53		0.02	3.71	8
	[14.54±2.51]	[-14.23±4.11]	[0.12±0.01]	[16.29±3.94]		[0.02±0.01]	[3.63±0.74]	[8.15±1.75]
13	20.03	-25.69	0.07	0.00	3.33		1.75	3
	[16.34±8.11]	[-16.95±19.32]	[0.08±0.02]	[0.11±0.33]	[3.26±0.50]		[2.46±1.65]	[4.92±2.84]
14	18.83	-22.73	0.10	0.00	3.30	0.04	2.78	7
	[17.60±5.51]	[-19.65±12.90]	[0.10±0.01]	[0.33±0.86]	[2.96±0.97]	[0.04±0.03]	[2.97±1.23]	[6.48±2.24]

Table S6. Testing mode and rates of morphological divergence of the two sister-genera *Pseudamnicola* and *Corrosella* along the MCC. We fitted two general types of models. The Brownian Motion (BM) model includes the rate of morphological divergence (σ^2) and the morphological optimum (θ). We used AIC-based model comparison to test additional variants of this model that allowed for genus and/or trait specific coefficients. Empty cells indicate identical coefficients for both trait axes or both genera, respectively. For each tested model, the first row indicates the coefficients and model fit of the MCC tree and the brackets in the second row include the average and standard deviation of these coefficients as well as the model fit based on 100 random post-burn-in trees. Because of the relatively small number of species, we could not fit more complex models with genus- and trait specific parameters or selection.

Model	ΔAIC	Mean rank	Genus	PCoA Axis 1		PCoA Axis 2	
				σ^2	θ	σ^2	θ
BM1	26.56	4	No genus differences	0.17	-0.03		-0.03
	[27.18±10.14]	[4.00±0.00]		[0.22±0.13]	[-0.03±0.00]		[-0.03±0.00]
BM2	17.04	3	No genus differences	0.26	-0.03	0.07	-0.03
	[17.42±7.22]	[3.00±0.00]		[0.36±0.23]	[-0.03±0.00]	[0.09±0.03]	[-0.03±0.00]
BM3	6.56	2	*Corrosella*	0.04	-0.15		-0.09
	[6.50±2.13]	[1.99±0.10]		[0.04±0.01]	[-0.16±0.02]		[-0.06±0.09]
			Pseudamnicola	0.26			
				[0.34±0.22]			
BM4	0.00	1	*Corrosella*	0.03	-0.15	0.05	-0.09
	[0.01±0.11]	[1.01±0.10]		[0.03±0.01]	[-0.15±0.03]	[0.05±0.01]	[-0.08±0.03]
			Pseudamnicola	0.43		0.09	
				[0.57±0.38]		[0.11±0.06]	
