# Supplementary material for: Ecological opportunity may facilitate diversification in Palearctic freshwater organisms: a case study on hydrobiid gastropods
Source: BMC Evol Biol. 2018 Apr 19;18:55. doi: 10.1186/s12862-018-1169-2 (PMC5907725; doi:10.1186/s12862-018-1169-2)

Supplementary material Figure S1: Phylogenetic relationships of *Corrosella* and *Pseudamnicola* species based on a Bayesian inference of the combined dataset. Bayesian posterior probabilities are indicated with black dots when <0.9. Bars on the right denote species assignments. For locality codes see Table S1.

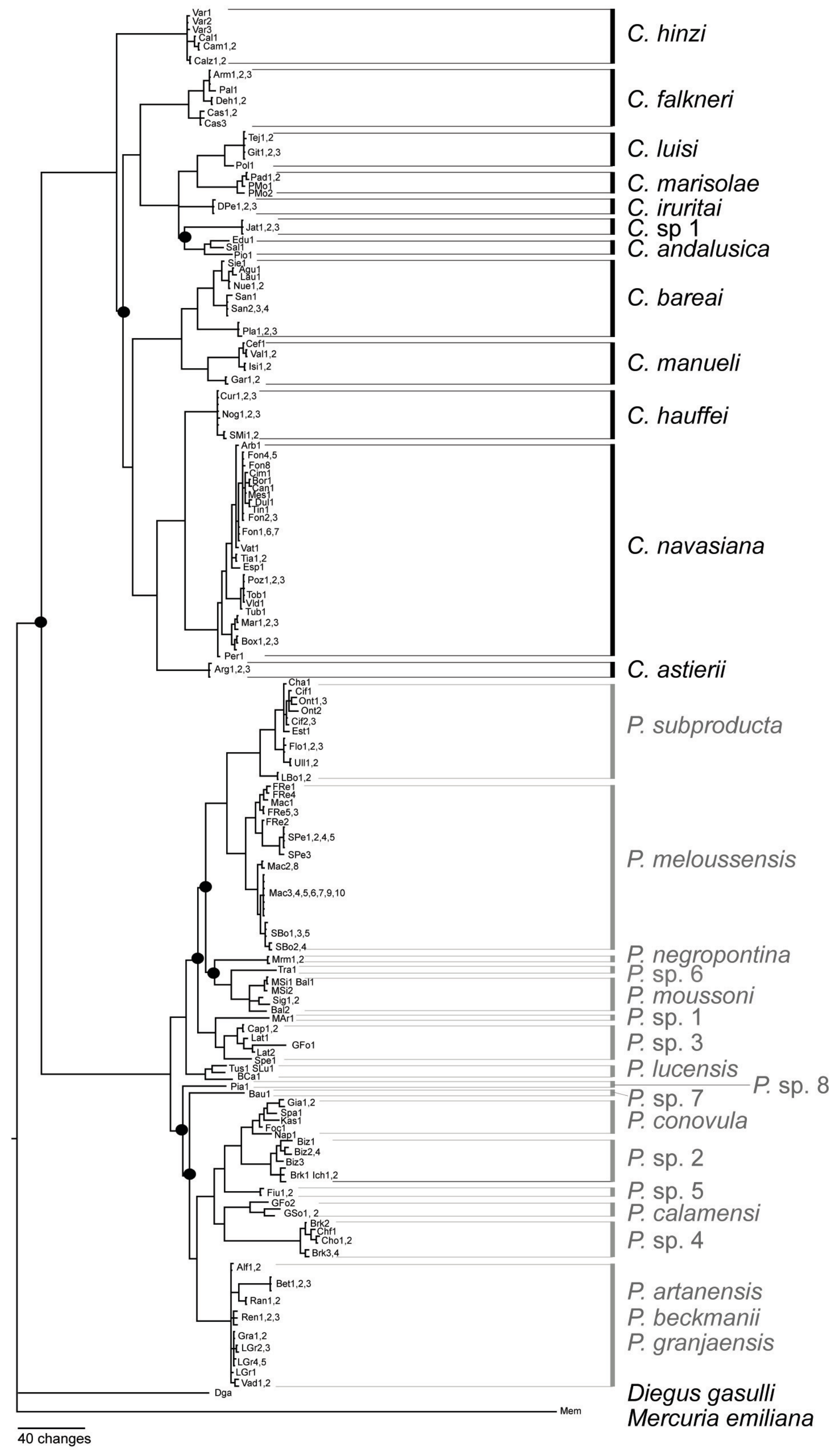

Supplement: Supplementary file 2 — Figure S1. Phylogenetic relationships of Corrosella and Pseudamnicola species based on a Bayesian inference of the combined COI, 16S, and 28S datasets. Bayesian posterior probabilities are indicated with black dots when < 0.9. Bars on the right denote species assignments. For locality codes see Additional file 1: Table S1. (PDF 2138 kb) [file 12862_2018_1169_MOESM2_ESM.pdf]
